# *Schizophyllum commune* Enhances Dendrobine Accumulation in *Dendrobium nobile* in Association with Induced Fungal Cellulolytic Activity and Host Jasmonate Biosynthesis

**DOI:** 10.3390/biom16101533

**Published:** 2026-10-20

**Authors:** Yongxia Zhao, Yonglin Ouyang, Nian Xiong, Xiaolong Ji, Qi Jia, Lin Qin, Jian Xie, Yuqi He

**Affiliations:** 1 State Key Laboratory of Discovery and Utilization of Functional Components in Traditional Chinese Medicine, Guizhou Medical University, Guiyang 561113, China; zhaoyongxia@zmu.edu.cn; 2 Guizhou Engineering Research Center of Industrial Key-Technology for Dendrobium Nobile, Guizhou Engineering Research Center for Orchid Medicinal Plant Breeding and Efficient Application, Zunyi Medical University, Zunyi 563000, China; ouyangyonglin@zmu.edu.cn (Y.O.); xiongnian@zmu.edu.cn (N.X.); jixiaolong@zmu.edu.cn (X.J.); jiaqi@zmu.edu.cn (Q.J.); qinlin@zmu.edu.cn (L.Q.); 3 School of Pharmacy, Guiyang Kangyang University, Guiyang 550081, China

**Keywords:** *Dendrobium nobile*, *Schizophyllum commune*, dendrobine, jasmonic acid signaling, cellulase, dual RNA seq

## Abstract

Objective: To explore the mechanism by which *Schizophyllum commune* elevates dendrobine accumulation in *Dendrobium nobile*, and verify whether cellulase derived from this fungus triggers jasmonate biosynthesis to mediate the regulation of alkaloid production. Methods: Endophytic fungi were isolated from *D. nobile* stems via tissue block isolation and identified by ITS sequencing. A plant–fungus symbiotic system was established with axenic plantlets. Dual RNA seq transcriptomic analysis, RT qPCR, pharmacological interventions with methyl jasmonate (MeJA) and the JA biosynthesis inhibitor salicylhydroxamic acid (SHAM) and Extracellular cellulase activities assays was performed. Results: In this study, none of 29 endophytic fungal strains from *D. nobile* stems produced dendrobine independently. Symbiotic screening subsequently identified *Schizophyllum commune* CY01 1H (SC) as the best strain that significantly increased dendrobine accumulation in *D. nobile* plantlets. Dual RNA seq revealed that SC colonization substantially activated jasmonic acid (JA) biosynthesis, accompanied by marked upregulation of the key biosynthetic gene DnLOX2. In contrast, downstream JA-signaling components exhibited an atypical transcriptional pattern, DnJAR was downregulated, whereas DnJAZ was upregulated. These results suggest that SC colonization induces a reconfiguration of JA signaling that may contribute to the maintenance of symbiotic homeostasis. In the fungus, cellulase-encoding genes were significantly induced. Experimental verification that MeJA promoted dendrobine accumulation, whereas SHAM suppressed it, and SC symbiosis partially mitigated this suppression. Stem section assays showed dynamic increases in extracellular cellulase activities (endoglucanase, cellobiohydrolase, β glucosidase), peaking at day 19, with endoglucanase being the highest. Conclusions: These findings support a cross-regulatory model in which *S. commune* CY01-1H induces cellulolytic enzyme production during colonization, coinciding with host JA-biosynthetic activation and JA-signaling reprogramming, ultimately enhancing dendrobine accumulation. This study provides integrated evidence from fungal and host transcriptomes and enzymatic assays, expanding our understanding of endophyte-mediated regulation of medicinal plant metabolism. *S. commune* CY01 1H represents a promising biostimulant for sustainable improvement of dendrobine yield in cultivated *D. nobile*.

## 1. Introduction

*Dendrobium nobile* Lindl. (*D. nobile*) is a perennial epiphytic orchid distributed predominantly in the subtropical regions of southern China, including Guizhou, Yunnan, and Hainan provinces. It is an important botanical source of “Shihu”, a highly valued traditional Chinese medicinal material. The medicinal properties of *D. nobile* are largely attributed to dendrobine, a characteristic bioactive alkaloid with immunomodulatory, anti-inflammatory, and neuroprotective activities [1], Dendrobine also serves as the principal quality marker for assessing the medicinal quality of *D. nobile*. Despite its considerable pharmacological and commercial value, the sustainable utilization of *D. nobile* is hindered by the depletion of wild resources and the unstable accumulation of dendrobine in cultivated and wild-simulated plants. Under these production conditions, dendrobine content is often low and varies substantially among plants, posing major challenges to quality control and the large-scale development of *D. nobile*-derived products. Our preliminary studies suggest that endophytic fungal communities associated with *D. nobile* may play an important role in regulating dendrobine biosynthesis and accumulation, thereby providing a potentially effective strategy to improve alkaloid production through targeted microbial manipulation [2].

Although our preliminary observations suggest a functional association between endophytic fungal communities and dendrobine accumulation in *D. nobile*, the specific microbial taxa involved and the regulatory pathways underlying this association remain largely unknown. Nevertheless, studies across diverse plant–microbe systems have established that beneficial microorganisms can profoundly influence host metabolism by modulating fatty acid metabolism and phytohormone-signaling networks [3,4]. These studies also highlight the mechanistic diversity of microbe-mediated metabolic regulation, encompassing phytohormone signaling, root exudate–microbiome interactions, and the induction of antimicrobial compounds. For example, the endophytic bacterium *Sphingomonas* enhances disease resistance in rice by promoting the accumulation of para-aminobenzoic acid [5]. In wheat and maize, root-exuded benzoxazinoids shape rhizosphere microbial communities and potentiate jasmonic acid (JA)-mediated signaling and defense responses [6]. *Streptomyces albidoflavus* CARA17 stimulates plant growth via antimicrobial compound induction [7], whereas *Burkholderia theae* GS2Y increases polyphenol accumulation in tea, thereby enhancing resistance to pathogens [8]. Finally, these findings demonstrate that beneficial microorganisms, through sustained interactions with their plant hosts, can act as important regulators of secondary metabolism and stress adaptation. However, whether comparable mechanisms contribute to dendrobine biosynthesis in the *D. nobile*-endophyte association, and which fungal taxa mediate these effects, remains to be determined.

Reports on endophytic fungi associated with *D. nobile* remain limited, and the functions of its diverse endophytic fungal community have yet to be fully characterized. In the present study, an endophytic fungal strain, *Schizophyllum commune* Fr.CY01-1H (SC), was isolated from *D. nobile* using the tissue-block method and identified through symbiotic screening as a potent promoter of dendrobine accumulation. *S. commune* belongs to the phylum Basidiomycota, order Agaricales, and family Schizophyllaceae. This fungus produces a range of bioactive metabolites, including fungal polysaccharides and sesquiterpenes, and has been associated with anticancer, anti-fatigue, and anti-inflammatory activities [9,10,11]. Notably, *S. commune* has also been reported to modulate secondary metabolism in host plants. For example, an *S. commune* strain isolated from *Ajuga bracteosa* promoted volatile-oil accumulation and enhanced antioxidant capacity in *Perilla frutescens* following inoculation [12]. In blueberry, *S. commune* increased flavonoid accumulation and contributed to the suppression of root rot [13]. Despite these observations, the molecular basis of the interaction between SC and *D. nobile remains* unknown. To address this question, we established an *S. commune*-*D. nobile* symbiotic system and employed dual RNA sequencing to simultaneously profile transcriptional changes in both partners. Dendrobine accumulation was further quantified by gas chromatography–mass spectrometry (GC-MS). Our results indicate that the SC-*D. nobile* interaction is associated with the induction of fungal cellulolytic activity and the activation and reprogramming of host jasmonic acid signaling, which may contribute to enhanced dendrobine accumulation.

## 2. Materials and Methods

### 2.1. Isolation, Purification and Fermentation of Endophytic Fungi of D. nobile

Fresh stems of *D. nobile* were surface-disinfected. After sterilization, the stems were cut into approximately 0.5 cm segments using a sterilized scalpel. The segments were placed on Martin medium plates, with the cut surfaces in close contact with the medium, and incubated at 28 °C for 3–7 days. Emerging fungal colonies were provisionally differentiated according to colony color and other macroscopic morphological characteristics. Hyphal tips from individual colonies were transferred to potato dextrose agar plates (PDA) with sterile inoculation needles and repeatedly subcultured until pure cultures were obtained. For morphological characterization, each purified isolate was inoculated onto PDA plates and incubated at 28 °C for 3–7 days. Colony morphology was recorded using an optical microscope and the adhesive-tape method. Preliminary identification was performed according to standard mycological manuals, and taxonomic identification was confirmed by internal transcribed spacer (ITS) region sequencing. Sequencing was conducted by Rhonin Biotechnology Co., Ltd. (Chengdu, China).

The 29 endophytic fungal isolates were revived and inoculated into potato dextrose broth (PDB) according to the grouping scheme shown in Table 1. Cultures were incubated at 28 °C on a rotary shaker at 200 rpm for 10 days. Following fermentation, cultures were centrifuged at 12,000 rpm for 10 min to separate the mycelial pellets from the culture supernatants. The supernatants were lyophilized under vacuum, whereas the mycelial pellets were oven-dried at 50 °C and ground into a fine powder. Both the lyophilized fermentation extracts and mycelial powders were subsequently analyzed for dendrobine content.

### 2.2. GC-MS Analysis of Dendrobine Content

Dendrobine was quantified by gas chromatography–mass spectrometry (GC-MS) using an Agilent DB-1MS UI capillary column (122-0132UI, 30 m × 0.250 mm × 0.25 μm, Agilent Technologies, Santa Clara, CA, USA). The injection volume was 1 μL with a split ratio of 100:1. The detector temperature was set to 250 °C; air flow was 300 mL/min, hydrogen flow 30 mL/min, makeup gas flow 25 mL/min, and carrier gas flow 1 mL/min. The oven temperature program began at 80 °C, increased to 250 °C at 10 °C/min, and was held for 5 min. A mixed standard solution containing 20.00 μg/mL dendrobine and 5.00 μg/mL naphthalene was prepared as the internal standard. The analysis was performed on a Shimadzu GCMS-TQ8040 NX triple-quadrupole gas chromatograph–mass spectrometer (Shimadzu Corporation, Kyoto, Japan).

Dried *D. nobile* plantlets, fungal mycelial pellets, and lyophilized fermentation extracts were ground into powder and sieved through a No. 3 sieve. For each sample, 0.25 g of powder was accurately weighed and extracted with 25 mL of 0.05% formic acid in methanol under reflux at 80 °C for 3 h. After cooling to room temperature, the extract was replenished to the original weight using the same extraction solvent and filtered. A 2-mL aliquot of the filtrate was transferred into a 5-mL volumetric flask, mixed with 1 mL of internal standard solution, and diluted to volume with methanol. The resultant solution was subjected to GC-MS analysis of dendrobine. Representative total-ion chromatograms and electron-impact mass spectra of the dendrobine reference standard and representative plant samples are provided in Supplementary Figure S2. Quantification was performed by the internal-standard method using naphthalene as the internal standard, with a calibration curve constructed from the dendrobine reference standard (R^2^ > 0.999); dendrobine contents are expressed on a dry-weight basis.

### 2.3. LC-MS/MS Quantification of Jasmonic Acid in D. nobile

Leaves and roots of *D. nobile* were separately ground into fine powder in liquid nitrogen. For each sample, 100 mg of powdered tissue was extracted with 0.6 mL of acetonitrile containing 1% formic acid via ultrasonication for 30 min, followed by centrifugation at 12,000 rpm for 10 min. The supernatant was retained, and the pellet was re-extracted using 0.4 mL of the same solvent. After vortexing, the suspension was ultrasonicated for another 30 min and centrifuged under identical conditions. Supernatants obtained from the two extraction steps were pooled and evaporated to dryness under a gentle nitrogen stream. The residue was reconstituted in 1 mL methanol, ultrasonicated for 30 min, and centrifuged at 12,000 rpm for 10 min. The final supernatant was collected for LC-MS/MS analysis, with three biological replicates prepared for each treatment.

Analyses were performed using an SCIEX liquid chromatography system coupled to an API 4000™ triple-quadrupole mass spectrometer (HPLC-QqQ-MS/MS; SCIEX, Framingham, MA, USA). Chromatographic separation was carried out on an ACQUITY UPLC BEH C18 column (1.7 μm, 2.1 × 50 mm; Waters Corporation, Milford, MA, USA) maintained at 30 °C, and the autosampler temperature was set to 15 °C. The mobile phase comprised 0.1% formic acid in water (A) and acetonitrile (B) at a flow rate of 0.2 mL/min, and the injection volume was 2 μL. Mass spectra were acquired on the API 4000™ system under negative electrospray ionization (ESI) mode with multiple reaction monitoring (MRM). Representative multiple-reaction-monitoring (MRM) chromatograms of the JA reference standard and representative plant samples are provided in Supplementary Figure S3.

### 2.4. Establishment of Plant–Fungus Symbiotic System and Dual RNA-Seq Transcriptomic Analysis

Once the plantlets were fully acclimated, a purified endophytic fungal isolate was inoculated onto the medium surface at the center of each culture bottle to avoid direct contact with the plantlets. The co-cultures were incubated in a growth chamber at 25 ± 1 °C under a 12 h photoperiod with a light intensity of 1500–2000 lx. Successful colonization was verified by visible mycelial extension toward the plantlets and microscopic observation of hyphae inside stem tissues, with no obvious adverse effects on plantlet growth. All plantlets were micropropagated from a uniform axenic stock established by asymbiotic germination of seeds collected from a single *D. nobile* plant at the seedling base, Chishui, Guizhou, China (105°48′50″ E, 28°28′26″ N), bulk-propagated plantlets were used at 3–4 cm in height. Each culture vessel contained five plantlets, and each treatment comprised five independent vessels as biological replicates, randomly assigned. The symbiotic medium was half-strength Murashige and Skoog (1/2 MS) medium supplemented with 30 g/L sucrose, 80 g/L potato extract, 0.5 mg/L α-naphthaleneacetic acid (NAA) and 5.5 g/L agar, pH 5.8. Because this isolate does not produce conidia under the tested conditions, 1 mg of freshly harvested 7-day-old PDB-grown mycelium was placed at the center of the medium surface, away from the plantlets. In addition to mycelial extension and microscopic observation, colonization was verified by a re-isolation assay: surface-disinfected stem segments of co-cultured plantlets were plated on PDA, and the fungus recovered was re-identified as *S. commune* by ITS sequencing. Dendrobine quantification, JA quantification, and dual RNA-seq were performed on the same 21-day co-cultivation samples (each vessel = one biological replicate, five plantlets pooled and uniformly ground), the MeJA/SHAM experiment described below was an independent parallel batch.

Samples of *D. nobile* plantlets colonized by *S. commune* and uninoculated sterile controls, together with pure fungal cultures grown in PDB medium, were collected with five biological replicates. All samples were flash-frozen in liquid nitrogen and stored at −80 °C before delivery to Novogene Co., Ltd. (Beijing, China) for dual RNA-seq analysis. Total RNA was extracted, and poly-A enrichment was applied to colonized plant samples to capture polyadenylated mRNAs from both host and fungus in a single library. After quality assessment, libraries were sequenced on an Illumina platform. Raw image data were converted into sequencing reads via CASAVA base calling, and quality filtering was performed to obtain clean reads for subsequent analysis. All clean reads were mapped against a combined reference genome containing genomic sequences of *D. nobile* and *S. commune*. Based on mapping results, reads were assigned to either plant or fungal origin. The separated datasets were then independently processed for gene expression quantification, differential expression analysis, and functional enrichment analysis. Sequencing was performed on an Illumina NovaSeq platform to generate 150 bp paired-end reads. The reference genomes used were the *D. nobile* genome assembly GCA_022539455.1 and the *S. commune* H4-8 genome assembly ADMJ00000000.2. Clean reads were mapped to the combined reference using HISAT2 (v2.1.0), and reads mapping ambiguously to both genomes were discarded. Gene expression was quantified as FPKM, and differentially expressed genes were identified using DESeq2 (v1.34.0) with thresholds of |log2 fold change| ≥ 1 and Benjamini–Hochberg-adjusted *p* < 0.05. KEGG enrichment was performed with clusterProfiler (v4.0.0) against the KEGG pathway database, using all expressed genes of each organism as the background universe. The raw sequencing data have been deposited in the NCBI Sequence Read Archive under a BioProject accession being finalized with NCBI curation; the accession and a private reviewer link will be provided to the editorial office and released upon publication.

### 2.5. Real-Time Quantitative PCR

Total RNA was extracted from *D. nobile* tissues using an RNA purification kit (TIANGEN, Beijing, China). RNA concentration and purity were assessed with a NanoDrop 2000 spectrophotometer (Thermo Fisher Scientific, Waltham, MA, USA). Reverse transcription was performed according to the manufacturer’s protocol using a reaction mixture containing the appropriate volumes of reagents and RNA, with the following cycling conditions: 42 °C for 40 min, 85 °C for 5 s, and then immediate cooling on ice. The resulting cDNA was stored at −20 °C. Gene-specific primers for target genes were designed using the NCBI Primer-BLAST tool (https://www.ncbi.nlm.nih.gov/tools/primer-blast/, accessed on 2 August 2026) based on the *D. nobile* genomic sequences and synthesized by Sangon Biotech (Shanghai, China). RT-qPCR was carried out in 20 μL reactions containing 2 × SYBR^®^ Green Supermix (TIANGEN, Beijing, China), cDNA template, and specific primers. The Dn*UBQ1* gene was used as the internal reference for normalization. Relative expression levels were calculated using the 2^−ΔΔCt^ method. RT-qPCR was reported in accordance with the MIQE guidelines [14]. The primer sequences, amplicon sizes and amplification efficiencies (90–110%) for all genes are listed in Supplementary Table S1; a single melt-curve peak confirmed amplification specificity. Dn*UBQ1* was used as the internal reference because its cycle threshold varied by <0.5 across axenic, colonized and MeJA/SHAM-treated samples, and because it has been validated as a reference gene in our previous *D. nobile* studies [2,3,4].

### 2.6. Co-Fermentation of S. commune with D. nobile Stem Sections

Fresh *D. nobile* stems were cut into small sections. Ten grams of stem sections were transferred into culture flasks containing 15 mL distilled water and autoclaved at 121 °C for 15 min. No free liquid remained in the flasks after sterilization, and the stem sections maintained moist conditions. Once cooled, 1 mL of *S. commune* seed culture was inoculated into each flask, with three biological replicates. Control flasks received 1 mL of sterile water in place of the fungal inoculum (three replicates). The flasks were incubated statically at 28 °C. Visible mycelial growth appeared on day 3, at which point flasks were gently shaken by hand to achieve uniform hyphal coverage on stem sections. Static fermentation proceeded for 10 days before sample collection for subsequent analyses.

### 2.7. Cellulase and Pectinase Activity Assays

Exo-β-1,4-cellobiohydrolase (CBH) activity was quantified using a commercial assay kit (Addison Biotechnology, Qingdao, China). Briefly, 0.1 g tissue was homogenized in 1 mL pre-chilled extraction buffer on ice. The homogenate was centrifuged at 12,000 rpm for 10 min at 4 °C, and the supernatant was retained on ice. A microplate reader was preheated for a minimum of 30 min, and absorbance was recorded at 405 nm.

Polygalacturonase (PG) and endo-β-1,4-glucanase (EG) activities were measured using matching assay kits from Addison Biotechnology. Tissue samples (0.2 g) were homogenized in 1 mL pre-chilled 95% ethanol on ice and kept at 4 °C for 10 min, followed by centrifugation at 12,000 rpm for 5 min at 4 °C. The supernatant was discarded, and the pellet was washed with 1 mL pre-chilled 80% ethanol. After vortexing and incubation at 4 °C for 10 min, the mixture was centrifuged again. The pellet was resuspended in 1 mL pre-chilled extraction buffer, vortexed and incubated at 4 °C for 10 min. Following centrifugation at 12,000 rpm for 10 min at 4 °C, the supernatant was kept on ice, and absorbance was detected at 540 nm.

β-Glucosidase (GC) activity was assayed with a kit supplied by Addison Biotechnology. Briefly, 0.1 g tissue was homogenized in 1 mL extraction buffer on ice and centrifuged at 12,000 rpm for 10 min at 4 °C. The supernatant was collected on ice, and absorbance was measured at 405 nm. Enzyme activities were calculated from standard curves supplied with the kits and are expressed as U per gram of fresh tissue. Uninoculated sterilized stem sections incubated under identical conditions served as blank controls at every time point, and their residual background activities were subtracted from the corresponding inoculated samples.

### 2.8. Pharmacological Treatments

To evaluate the contribution of JA to dendrobine accumulation, axenic and SC-colonized plantlets were treated with MeJA 50 μmol/L or the JA-biosynthesis inhibitor SHAM 500 μmol/L. A 2 mL aliquot of each solution was applied once by aseptic spraying onto the medium surface on the day of inoculation (day 0), control plantlets received an equal volume of sterile water. Doses were selected from the published range used to manipulate JA in plant systems. Six groups (CK, SC, MeJA, SHAM, SC + MeJA, SC + SHAM) were cultivated and harvested in parallel, *n* = 5 independent vessels per group. No visible phytotoxic symptoms (chlorosis, necrosis or growth inhibition) were observed at these concentrations.

### 2.9. Statistical Analysis

All experiments were performed with independent biological replicates as specified for each experiment. Data are presented as the mean ± standard deviation (SD). Normality and homogeneity of variances were verified using the Shapiro–Wilk and Levene tests, respectively. Pairwise comparisons were evaluated by two-tailed Student’s *t*-test, and comparisons among multiple groups were evaluated by one-way analysis of variance (ANOVA) followed by Tukey’s honestly significant difference (HSD) post hoc test; *p* < 0.05 was considered statistically significant.

## 3. Results

### 3.1. Isolation and Taxonomic Characterization of 29 Endophytic Fungi from D. nobile Stems

Our previous work has shown that *D. nobile* hosts a diverse endophytic fungal community, and the abundance and taxonomic composition of these endophytes are tightly linked to the medicinal quality of the host plant [4]. To further characterize this microbial resource, we adopted the tissue segment method to isolate endophytic fungi from stems of wild-simulated *D. nobile*. A total of 63 fungal isolates were obtained. After repeated purification and identification based on colony morphology and internal transcribed spacer (ITS) sequences, these isolates were classified into 29 fungal taxa (Table 2). At the phylum level, 25 isolates belonged to Ascomycota and four to Basidiomycota. At the class level, the isolates were distributed across Sordariomycetes (15 isolates), Dothideomycetes (7 isolates), Agaricomycetes (4 isolates), and Eurotiomycetes (3 isolates).

The 29 fungal taxa were divided into five groups according to their taxonomic affiliations for shake-flask fermentation, as outlined in Table 1. Subsequent GC–MS analysis failed to detect dendrobine in either mycelial pellets or fermented culture supernatants (Supplementary Figure S1). These results indicate that none of the tested isolates synthesized detectable dendrobine under the applied fermentation conditions. Therefore, dendrobine accumulation in *D. nobile* is unlikely to arise solely from direct fungal biosynthesis.

### 3.2. Screening and Morphology of Candidate Endophytic Fungi

To screen endophytic fungi with beneficial effects on *D. nobile*, we reviewed published literature for the 29 obtained isolates and summarized their documented interactions with plant hosts (Table 3). *Bjerkandera adusta* inhibits the pathogen causing cabbage-type rapeseed wilt after colonizing cabbage and serves as a potential biocontrol agent for this wilt disease [15]. This fungus also suppresses growth of the wheat pathogen *Fusarium graminearum*, reducing the incidence of Fusarium head blight [16]. As a rice endophyte, *Preussia* sp. promotes host growth by synthesizing nitric oxide, indole-3-acetic acid, and gibberellins, including GA4, GA7, GA15, and GA53 [17]. Under drought stress, *Preussia* sp. improves total biomass, root biomass and active compound accumulation in *Glycyrrhiza uralensis* [18]. *S. commune*, a rare edible and medicinal basidiomycete, produces abundant crude enzymes and polysaccharides. Previous reports show that it promotes volatile oil accumulation in *Perilla frutescens* and enhances antioxidant capacity [12]. It also stimulates flavonoid accumulation in blueberry and effectively suppresses root rot disease [13]. *Trichoderma guizhouense* effectively controls anthracnose in oil tea [19] and when applied to maize and watermelon, promotes seed germination, improves plant growth, and enhances rhizosphere soil quality [20]. *Hypoxylon fragiforme* can strengthen plant resistance against anthracnose pathogens [21].

**Figure 1:**
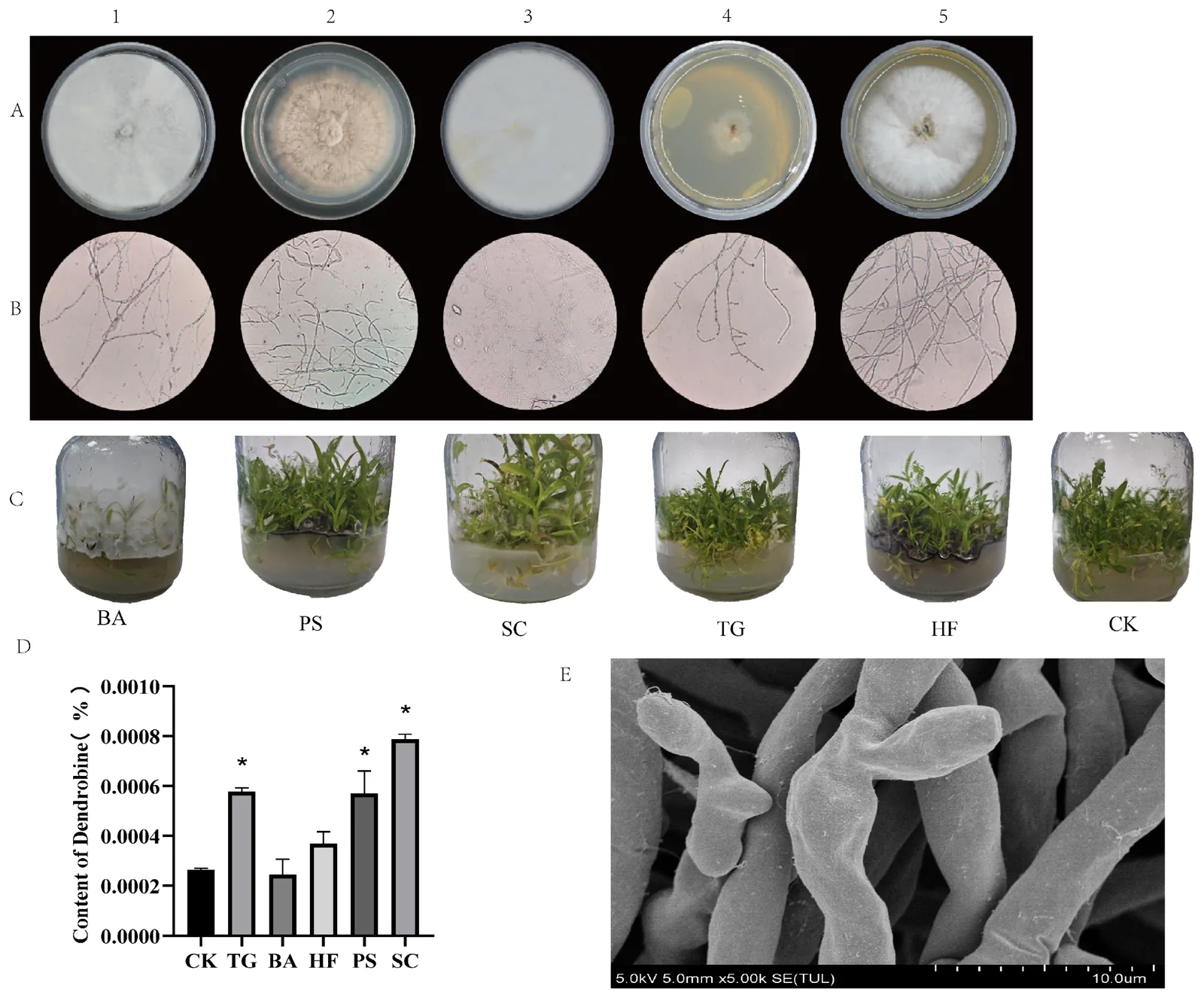
Screening of beneficial endophytic fungi and establishment of a symbiotic system with *D. nobile*. Five fungal isolates were evaluated: (1) *Bjerkandera adusta* CY03-4E (BA), (2) *Preussia* sp. HSS-1C (PS), (3) *S. commune* CY01-1H (SC), (4) *Trichoderma guizhouense* YJ01-10H (TG), and (5) *Hypoxylon fragiforme* YJ02-1B (HF). (**A**) Colony morphology of the five isolates grown on potato dextrose agar (PDA) medium. (**B**) Light micrographs showing the microscopic characteristics of the fungal isolates (100× magnification). (**C**) Phenotypic appearance of *D. nobile* plantlets after 21 days of co-cultivation with the respective fungal isolates. CK, uninoculated axenic control. (**D**) Dendrobine content in *D. nobile* plantlets after 21 days of co-cultivation. Asterisks indicate significant differences relative to the CK group (*p* < 0.05). (**E**) Scanning electron micrograph of *S. commune* CY01-1H hyphae showing clamp connections and extracellular globular secretions; no fruiting bodies or spore-bearing structures were observed.

On the basis of the endophytic fungal community analysis of stems from wild-simulated *D. nobile* and the reported biological activities of the isolates (Table 3), five strains were selected for the establishment of plant–fungus symbiotic systems with *D. nobile*. The selected strains included two basidiomycetes, *B. adusta* CY03-4E (BA) and *S. commune* CY01-1H (SC), as well as three ascomycetes, *Preussia* sp. HSS-1C (PS), *T. guizhouense* YJ01-10H (TG), and *H. fragiforme* YJ02-1B (HF) (Figure 1). The candidate isolates were further characterized by microscopic morphological features. *B. adusta* CY03-4E (BA), a Basidiomycota member, forms slender, flattened, hyaline, septate and wrinkled hyphae with limited branching during its anamorphic stage. Asexual reproduction occurred through hyphal fragmentation. *Preussia* sp. HSS-1C (PS), belonging to the phylum Ascomycota, produced hyaline to pale, septate, sparsely branched hyphae and colorless, unicellular, ellipsoidal ascospores. *S. commune* CY01-1H (SC), a basidiomycetous fungus, exhibited hyaline, septate, highly branched hyphae and colorless, smooth-surfaced, ellipsoidal spores. *T. guizhouense* YJ01-10H (TG), an ascomycetous fungus, developed profusely branched, hyaline, septate hyphae. Conidiophores arose terminally or laterally from the hyphae and were flask-shaped, producing unicellular, colorless, spherical to ellipsoidal, smooth-surfaced conidia. *H. fragiforme* YJ02-1B (HF), also belonging to the phylum Ascomycota, produced hyaline to light-brown, septate, sparsely branched hyphae and colorless, ellipsoidal ascospores (Figure 1A,B).

When axenic *D. nobile* plantlets grew to 3–4 cm in height, the candidate endophytic fungi were cultured in potato dextrose broth (PDB) at 28 °C for 7 days to collect mycelial biomass. The harvested mycelia were inoculated onto the surface of the symbiotic medium without direct contact with the *D. nobile* plantlets. The co-cultures were incubated in a controlled growth chamber at 25 ± 1 °C under a 12 h photoperiod with a light intensity of 1500–2000 lx for 3 weeks. During co-cultivation, fungal colony expansion toward the plantlets was observed, and hyphae were detected within the stems, while the plantlets remained healthy and continued to grow normally. These observations confirmed the successful establishment of the endophyte–*D. nobile* co-culture system.

After three weeks of co-cultivation, the plantlets inoculated with *Bjerkandera adusta* CY03-4E (BA) exhibited restricted growth, extensive leaf yellowing, and partial mortality compared with the axenic control plantlets (Figure 1C). In contrast, the plantlets in the HF, TG, and SC groups showed no apparent growth inhibition, and neither stem height nor stem diameter differed significantly from those of the axenic controls. GC-MS analysis of plant samples revealed that dendrobine levels were higher in the SC, PS, and TG groups than in the axenic controls. Among these treatments, SC exerted the strongest promoting effect, increasing dendrobine accumulation to 2.99-fold that of the control (*p* < 0.05, Figure 1D). Accordingly, *S. commune* CY01-1H was selected as the most effective strain for promoting dendrobine accumulation in *D. nobile*.

*S. commune* CY01-1H belongs to the phylum Basidiomycota, class Agaricomycetes, order Agaricales, family Schizophyllaceae, and genus *Schizophyllum*. Its colonies consisted of white, loosely textured mycelia with radially wrinkled surfaces and regular margins. Under light microscopy, the hyphae were hyaline, septate, and highly branched. Scanning electron microscopy further revealed clamp connections at the septa, short, curved, peg-like lateral projections, and tear-drop-shaped globular secretions on the hyphal surface. No fruiting bodies or spore-bearing structures were observed under the tested conditions (Figure 1E).

### 3.3. S. commune CY01-1H Colonization Is Associated with Activation of Jasmonic Acid Metabolism and Signaling in D. nobile

Although establishment of the *S. commune*-*D. nobile* symbiotic system substantially increased dendrobine accumulation, the molecular basis of this effect remained unclear. Therefore, five biological replicates of *D. nobile* plantlets successfully colonized by *S. commune* CY01-1H, exhibiting normal growth and confirmed fungal establishment, were selected for dual RNA-seq analysis. Five biological replicates each of axenic *D. nobile* plantlets and pure *S. commune* cultures were included as controls. Samples were separately labeled, immediately frozen in liquid nitrogen, and subjected to dual RNA-seq analysis. Following sequencing, raw data were filtered and evaluated for sequencing error rate, base-quality distribution, and GC content to obtain high-quality clean reads for downstream analyses. The number of clean reads per library ranged from 47,819,722 to 90,672,066. All libraries had sequencing error rates below 0.01%, Q20 values above 97.73%, Q30 values above 93.51%, and GC contents ranging from 43.39% to 52.99%. These metrics indicated that the sequencing data were of sufficient quality for subsequent analyses.

**Figure 2:**
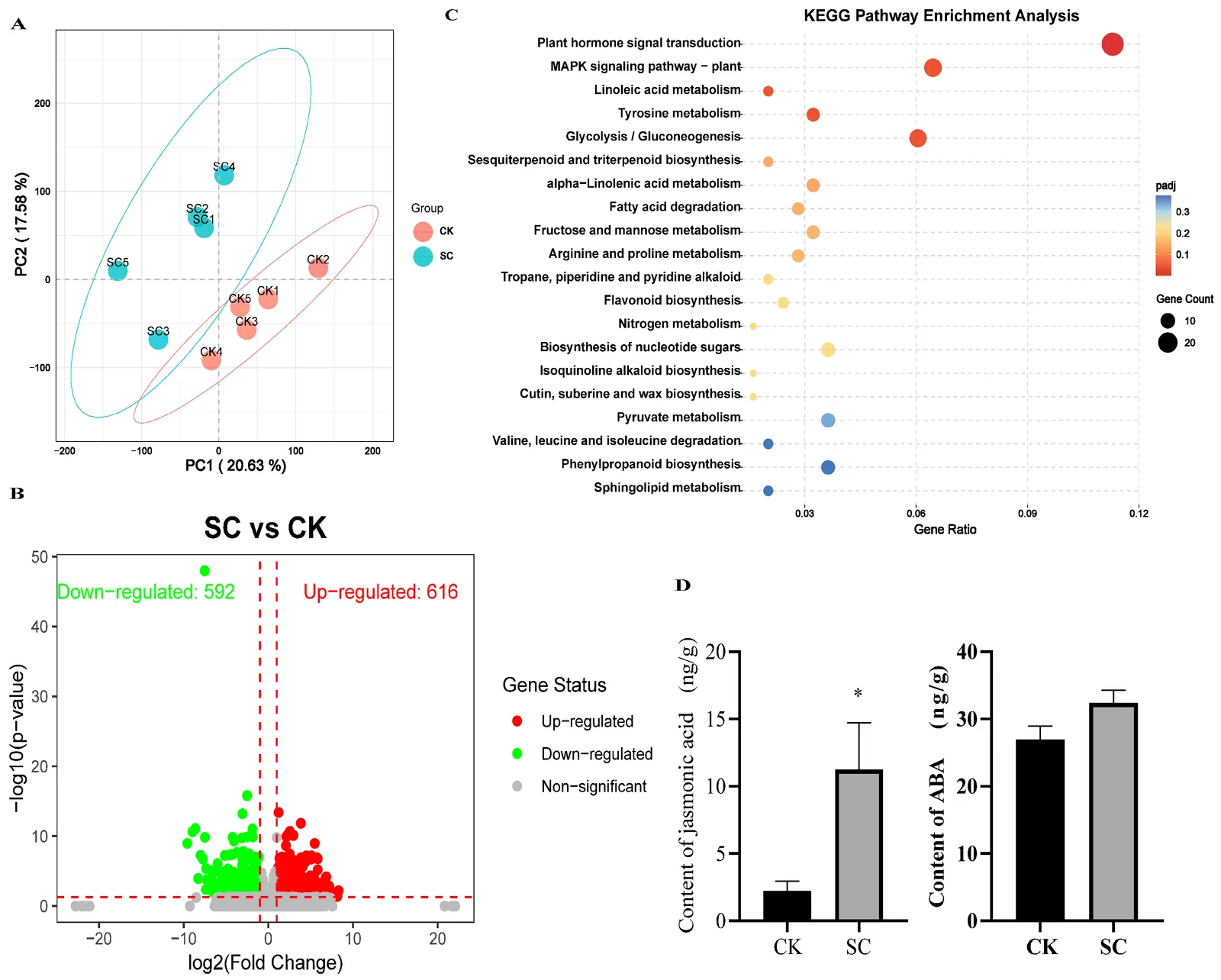
Dual RNA-seq analysis of *D. nobile* plantlets colonized by *S. commune* CY01-1H. (**A**) Principal component analysis of host transcriptomes from axenic control plantlets (CK) and *S. commune*-colonized plantlets (SC). (**B**) Volcano plot of differentially expressed genes (DEGs) between SC and CK plantlets, showing 616 upregulated and 592 downregulated genes. (**C**) KEGG pathway-enrichment analysis of the 1208 DEGs identified between SC and CK plantlets. The 20 most significantly enriched pathways are shown, including the plant hormone signal-transduction pathway. (**D**) Jasmonic acid (JA) and abscisic acid (ABA) contents in CK and SC plantlets. Data are presented in ng/g. Asterisks indicate significant differences between treatments (*p* < 0.05).

At the host-transcriptome level, RNA-seq detected 25,323 genes in *D. nobile*. PCA based on FPKM values was performed to assess variation among treatments and reproducibility among biological replicates. PC1 and PC2 explained 20.63% and 17.59% of the total variance, respectively. The axenic control (CK) and *S. commune*-colonized (SC) samples formed distinct clusters, indicating substantial transcriptional differences between the two groups (Figure 2A). To identify differentially expressed genes (DEGs) in both the host and fungal partners, comparisons were performed between SC-colonized and axenic *D. nobile* plantlets, as well as between *S. commune* in pure culture and during symbiosis. DEGs were defined using thresholds of ∣log_2_fold change∣ ≥ 1 and adjusted *p* value < 0.05. In the host comparison, 1208 DEGs were identified between SC-colonized and axenic plantlets, including 616 upregulated and 592 downregulated genes (Figure 2B).

Endophytic fungi can act as biostimulants by activating hormone-signaling pathways in plants and thereby regulating host growth, development, and metabolite accumulation. To investigate the metabolic pathways associated with the DEGs potentially involved in *S. commune*-mediated regulation of dendrobine accumulation in *D. nobile*, we performed KEGG pathway-enrichment analysis of the DEGs identified between SC-colonized and axenic plantlets. Among the 1208 DEGs detected in the SC-versus-CK comparison, the 20 most significantly enriched pathways, ranked according to adjusted *p* values, are shown in Figure 2C. Notably, the plant hormone signal-transduction pathway was among the pathways potentially associated with the response to endophytic fungal colonization and dendrobine accumulation. A total of 28 DEGs were assigned to the plant hormone signal-transduction pathway. These genes were distributed among seven hormone-signaling pathways, including those of auxin, cytokinins, gibberellins, abscisic acid (ABA), ethylene, brassinosteroids, and jasmonic acid (JA). Seven DEGs were enriched in the ABA-signaling pathway, whereas five were assigned to the JA-signaling pathway. We therefore further quantified ABA and JA levels in *D. nobile* plantlets (Figure 2D). ABA levels did not differ significantly between the SC and CK groups (*p* > 0.05), whereas JA levels were significantly higher in SC-colonized plantlets than in axenic controls (*p* < 0.05). These results suggest that SC colonization preferentially activates JA accumulation rather than ABA accumulation in *D. nobile*.

**Figure 3:**
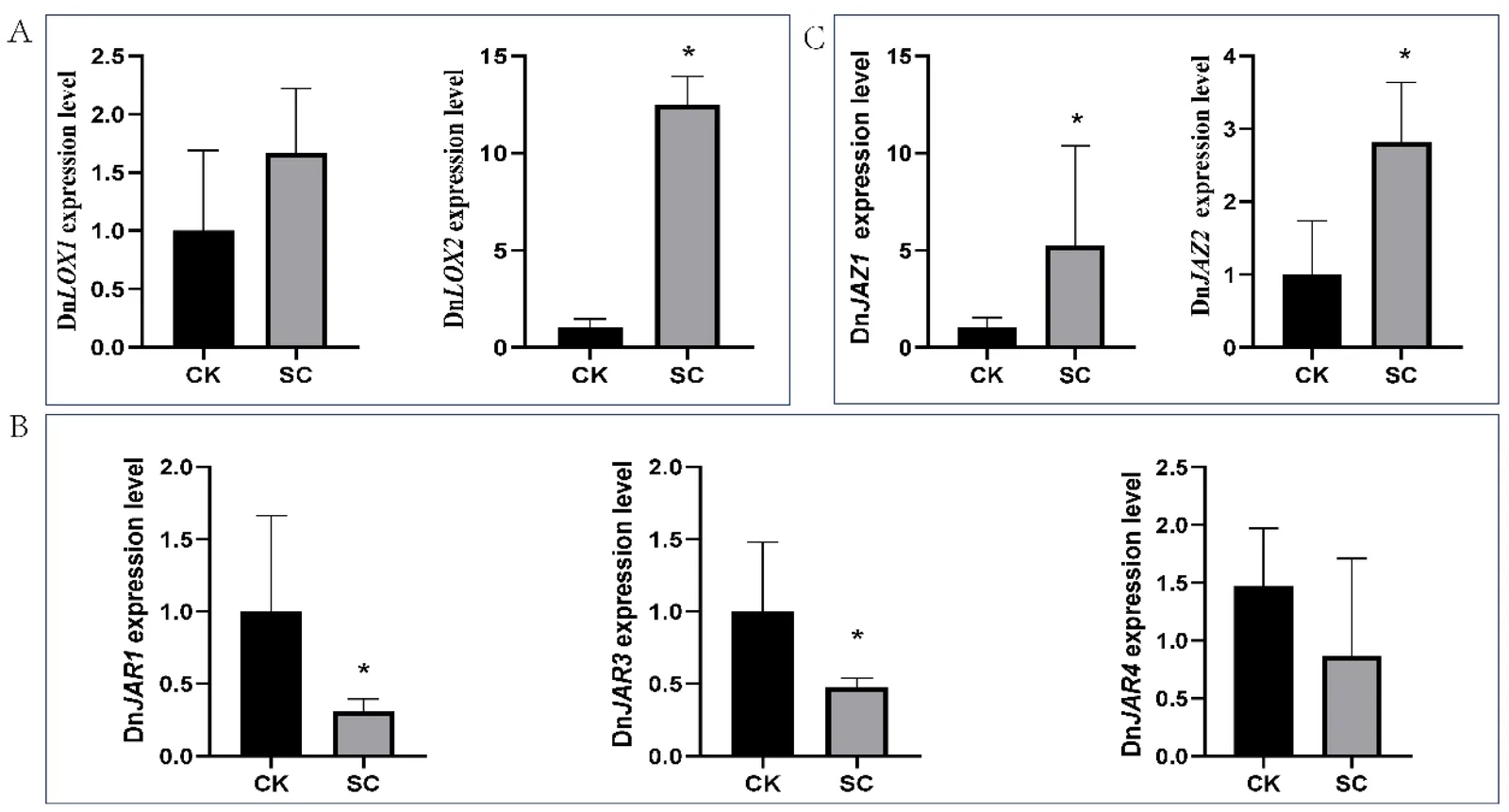
Differential expression of genes involved in jasmonic acid biosynthesis and signaling in axenic and *S. commune*-colonized *D. nobile* plantlets. (**A**) RT-qPCR validation of the differential expression of Dn*LOX1* and Dn*LOX2* between axenic control (CK) and *S. commune*-colonized (SC) plantlets. (**B**) RT-qPCR validation of the differential expression of Dn*JAR1*, Dn*JAR3*, and Dn*JAR4* between CK and SC plantlets. (**C**) RT-qPCR validation of the differential expression of Dn*JAZ1* and Dn*JAZ2* between CK and SC plantlets. Asterisks indicate significant differences between treatments (*p* < 0.05).

Consistent with previous reports, colonization by endophytic fungi can alter JA levels in host plants. Because jasmonate biosynthesis is initiated through the α-linolenic acid metabolic pathway, we further examined the α-linolenic acid metabolism and JA-signaling pathways. Key genes in both pathways showed an overall upregulation trend following colonization of *D. nobile* by *S. commune*. This pattern was confirmed by RT-qPCR, which showed that expression of the JA-biosynthetic gene Dn*LOX2* increased 11.96-fold in SC-colonized plantlets, Dn*LOX1* also showed an upward trend (Figure 3C). *DnLOX1* and *DnLOX2* encode lipoxygenases (LOXs), which catalyze the first committed step of the JA biosynthesis pathway. These results indicate that *S. commune* colonization is associated with enhanced JA biosynthesis in *D. nobile*.

In contrast, the downstream JA-signaling components exhibited a distinct transcriptional response. Expression of the JA-conjugating enzyme genes *DnJAR1* and *DnJAR3* was significantly reduced (Figure 3B), whereas the expression of genes encoding *DnJAZ* proteins showed an increasing trend (Figure 3C). The concomitant activation of JA biosynthesis and attenuation of downstream JA signaling suggests that *S. commune* does not elicit a strong, sustained immune response in *D. nobile*. Instead, the fungus may induce a controlled or reprogrammed JA response that maintains host immune readiness while preventing excessive defense activation that could compromise the symbiotic association. Such a balanced defense state is characteristic of compatible plant–microbe interactions and may provide a regulatory link between fungal colonization and host secondary metabolism [39], Collectively, these findings suggest that colonization by *S. commune* stimulates JA biosynthesis and modifies the transcriptional regulation of JA signaling in *D. nobile*, thereby potentially contributing to the enhanced accumulation of dendrobine.

### 3.4. Fungal Carbohydrate Metabolism and Cellulase Biosynthesis Are Enhanced During S. commune–D. nobile Symbiosis

**Figure 4:**
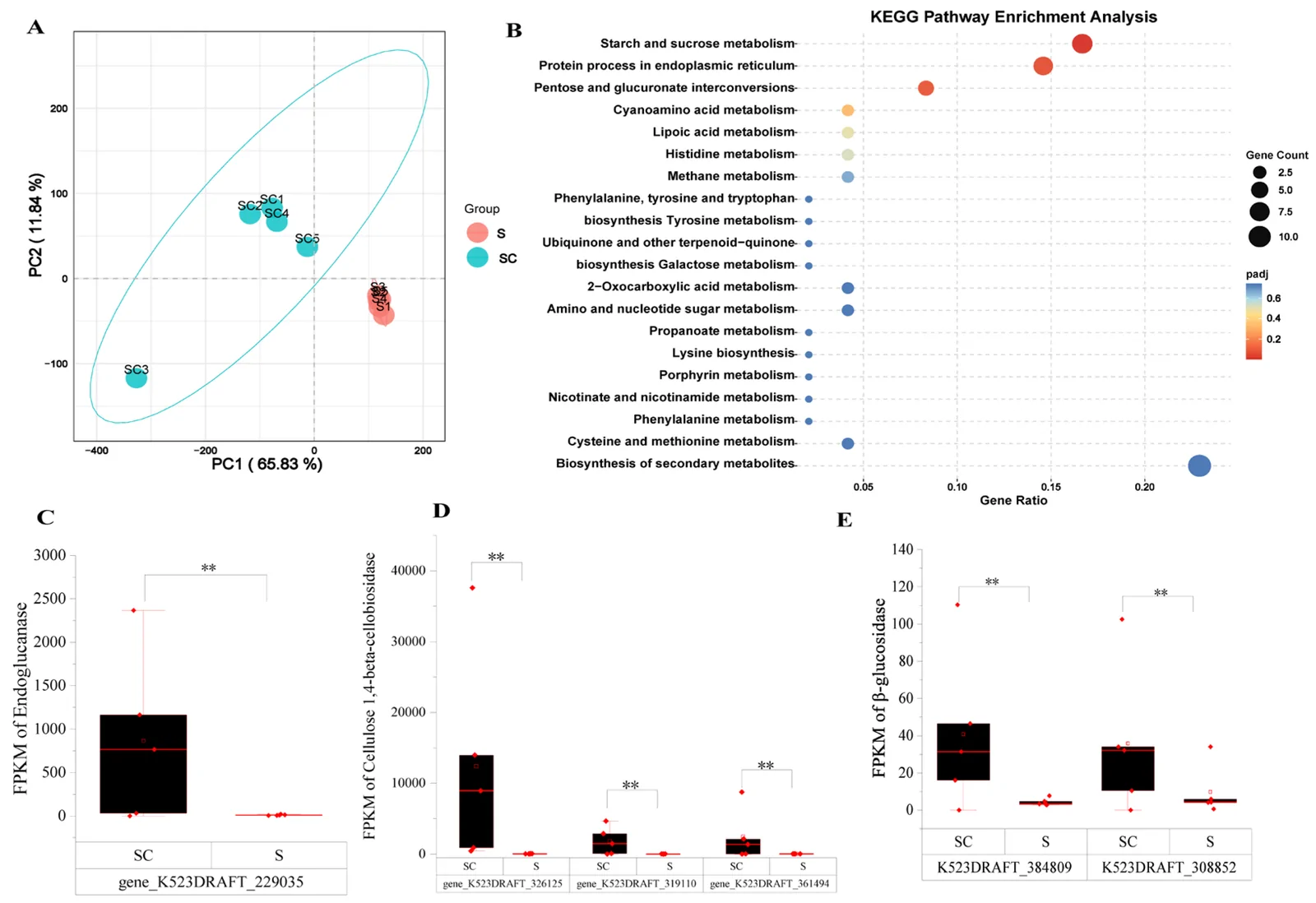
Dual RNA-seq analysis of *S. commune* CY01-1H during interaction with *D. nobile* plantlets. (**A**) Principal component analysis of *S. commune* CY01-1H grown in pure culture (S) and in association with *D. nobile* plantlets (SC). (**B**) KEGG pathway-enrichment analysis of the 359 differentially expressed genes (DEGs) identified between the SC and S groups. The 20 most significantly enriched pathways are shown, including the starch and sucrose metabolism pathway. (**C**) Differential expression of the endoglucanase-encoding gene *gene_K523DRAFT_229035* between pure-culture and symbiotic *S. commune* CY01-1H (*p* < 0.01). (**D**) Differential expression of the cellulose 1,4-β-cellobiosidase-encoding genes *gene_K523DRAFT_326125*, *gene_K523DRAFT_319110*, and *gene_K523DRAFT_361494* between pure-culture and symbiotic *S. commune* CY01-1H (*p* < 0.01). (**E**) Differential expression of the β-glucosidase-encoding genes *gene_K523DRAFT_384809* and *gene_K523DRAFT_308852* between pure-culture and symbiotic *S. commune* CY01-1H (*p* < 0.01). Red dots represent individual biological replicates. Two asterisks indicate *p* < 0.01.

While the host transcriptional responses were presented in Section 3.3, here we focus on the fungal counterpart to obtain a complete picture of the bidirectional interaction. Dual RNA-seq detected 16,963 genes in *S. commune*. PCA based on FPKM values was performed to assess transcriptional variation among treatments and the reproducibility of biological replicates. PC1 explained 65.83% of the total variance. The samples from *S. commune* colonizing *D. nobile* (SC group) were clearly separated from those of *S. commune* grown in pure culture (S group), indicating substantial transcriptional reprogramming of the fungus during host association (Figure 4A). Using ∣log_2_fold change∣ ≥ 1 and adjusted *p* value (*padj*) < 0.05 as the screening criteria, 359 differentially expressed genes (DEGs) were identified in the symbiotic fungus relative to the pure-culture fungus. Among these DEGs, 131 were upregulated and 228 were downregulated. KEGG pathway-enrichment analysis showed that the starch and sucrose metabolism pathway was significantly enriched (Figure 4B). Six genes in this pathway were significantly more highly expressed in the symbiotic fungus than in the pure-culture control (*p* < 0.01) and were primarily associated with the conversion of cellulose to D-glucose.

Endoglucanases randomly hydrolyze internal β-1,4-glycosidic bonds within the cellulose chain and are key enzymes involved in the initial conversion of cellulose into cellodextrins. In the symbiotic fungus, the expression of the endoglucanase-encoding gene *gene_K523DRAFT_229035* was significantly higher than that in the pure-culture fungus (Figure 4C). Cellodextrins can subsequently be hydrolyzed to cellobiose by cellulose 1,4-β-cellobiosidase. Depending on the structure of the cellulose substrate, cellulose may also be converted directly to cellobiose by this enzyme. Cellulose 1,4-β-cellobiosidases primarily hydrolyze β-1,4-glycosidic bonds sequentially from the nonreducing ends of cellulose chains or cellooligosaccharides, releasing cellobiose. The genes *gene_K523DRAFT_326125*, *gene_K523DRAFT_319110*, and *gene_K523DRAFT_361494*, which encode cellulose 1,4-β-cellobiosidases, were expressed at levels more than 1000-fold higher in the SC group than in the S group (Figure 4D).

Finally, β-glucosidases hydrolyze cellobiose and soluble cellooligosaccharides to glucose. The FPKM values of the β-glucosidase-encoding genes *gene_K523DRAFT_384809* and *gene_K523DRAFT_308852* were also significantly higher in the SC group than in the S group (Figure 4E). These results indicate that association with *D. nobile* stimulates the expression of a coordinated set of fungal cellulolytic enzymes, including endoglucanases, cellobiohydrolases, and β-glucosidases. The induction of these enzyme classes suggests enhanced activation of the cellulolytic pathway, enabling the progressive and potentially complete degradation of cellulose to glucose during symbiotic interaction. This fungal transcriptional response provides important mechanistic evidence for the role of carbohydrate metabolism and cell-wall-degrading enzymes in the *S. commune*–*D. nobile* association.

Collectively, these transcriptional data demonstrate that symbiotic interaction with *D. nobile* activates the complete cellulolytic machinery of *S. commune* CY01-1H, encompassing endoglucanases, exoglucanases (cellobiohydrolases), and β-glucosidases. This coordinated upregulation of the entire cellulose-to-glucose conversion pathway provides molecular evidence consistent with an enhanced capacity of the fungus for cell wall degradation during host association. Such a response may facilitate fungal invasion and nutrient acquisition, while simultaneously contributing to host cell wall remodeling—an event that may serve as an upstream trigger for the activation of plant defense signaling and secondary metabolism, as observed in the host transcriptome (Section 3.3).

### 3.5. Host Jasmonate Signaling and Fungal Cellulolytic Activity Are Associated with Enhanced Dendrobine Accumulation During the S. commune–D. nobile Interaction

To validate the transcriptomic results, key genes involved in α-linolenic acid metabolism and jasmonate (JA)-related signaling were examined by RT-qPCR. Following colonization by *S. commune*, both Dn*LOX1* (*KFK09_024652*) and *DnLOX2* (*KFK09_024653*) showed increased expression. In particular, Dn*LOX2* expression increased by 11.96-fold (*p* < 0.05), whereas Dn*LOX1* expression increased by 1.21-fold (Figure 3A). These results support the transcriptional activation of the α-linolenic acid pathway during fungal colonization, consistent with enhanced JA biosynthesis.

**Figure 5:**
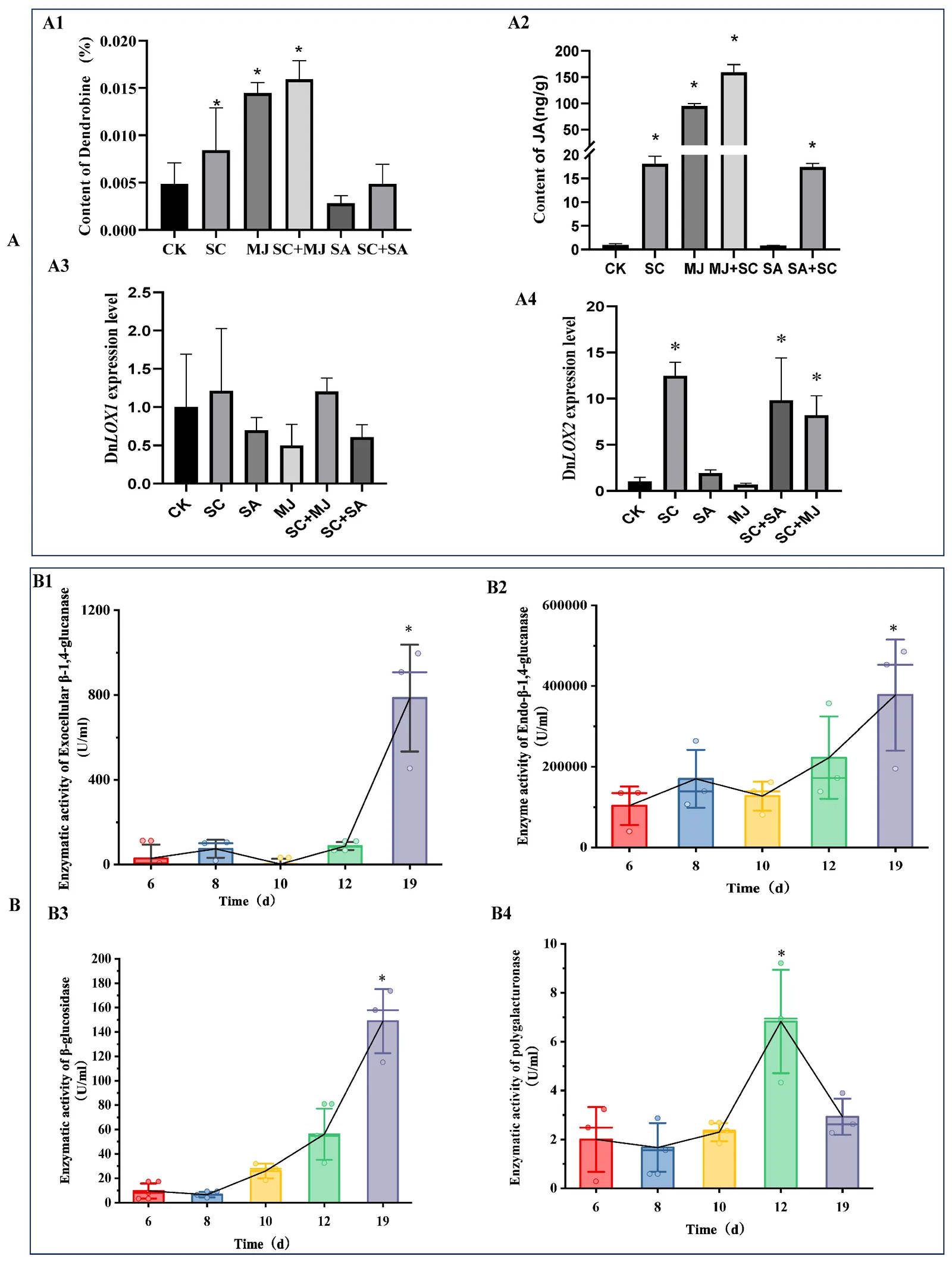
Validation of JA signaling and fungal cellulolytic activity in the *Dendrobium nobile*–*Schizophyllum commune* CY01-1H symbiotic system. (**A**) Pharmacological validation of the involvement of the JA pathway in the SC-mediated enhancement of dendrobine accumulation. Treatment groups were defined as follows: CK, axenic plantlets treated with sterile water; SC, *S. commune* CY01-1H-colonized plantlets; MeJA, axenic plantlets treated with 2 mL of 50 μmol/L methyl jasmonate; SHAM, axenic plantlets treated with 2 mL of 500 μmol/L salicylhydroxamic acid, an inhibitor of JA biosynthesis; SC + MeJA, *S. commune* CY01-1H-colonized plantlets treated with 2 mL of 50 μmol/L MeJA; and SC + SHAM, *S. commune* CY01-1H-colonized plantlets treated with 2 mL of 500 μmol/L SHAM. (**A1**) Dendrobine content. (**A2**) Endogenous JA content. (**A3**,**A4**) Relative expression levels of Dn*LOX1* and Dn*LOX2*, respectively, across the six treatment groups. (**B**) Time-course analysis of cellulolytic enzyme activities produced by *S. commune* CY01-1H during co-culture with *D. nobile* stem sections from days 6 to 19. (**B1**) Exo-β-1,4-cellobiohydrolase (CBH) activity. (**B2**) Endo-β-1,4-glucanase (EG) activity. (**B3**) β-Glucosidase (BG) activity. (**B4**) Polygalacturonase (PG) activity. Asterisks indicate statistically significant differences between the indicated groups or time points (*p* < 0.05).

To further examine the contribution of JA to dendrobine accumulation, JA contents were quantified in *S. commune*-colonized *D. nobile* plantlets (SC). JA content in the SC group reached 18.09 ng/g (mean ± SD, *n* = 3), which was significantly higher than that in axenic control plantlets (CK) (Figure 5(A2)). Application of exogenous MeJA significantly increased dendrobine content in axenic plantlets. Moreover, MeJA treatment of SC-colonized plantlets (SC + MeJA) further enhanced dendrobine accumulation relative to that in the SC treatment alone (Figure 5(A1)). Conversely, treatment of SC-colonized plantlets with salicylhydroxamic acid (SHAM), an inhibitor of JA biosynthesis, significantly reduced both JA and dendrobine contents (Figure 5(A1,A2)). Expression analysis of Dn*LOX1* and Dn*LOX2* in the CK, SC, MeJA, SHAM, SC + MeJA, and SC + SHAM groups showed patterns consistent with the changes in JA levels and dendrobine accumulation (Figure 5(A3,A4)). Together, these results indicate that JA is an important regulatory component of *S. commune*-mediated dendrobine accumulation in *D. nobile*. Although pharmacological inhibitors are never perfectly selective, the SC + SHAM group showed that SC colonization partially relieved the SHAM-driven suppression of both JA and dendrobine, which is consistent with a contribution of JA to the fungal effect.

The marked induction of cellulase-encoding genes in symbiotic *S. commune* prompted us to further determine cellulolytic enzyme activities during fungal interaction with *D. nobile* stem tissues. *S. commune* was inoculated onto sterilized *D. nobile* stem sections without the addition of an external nutrient source, and enzyme activities were monitored during static incubation at 28 °C. Cellulolytic activities became consistently detectable after 6 days of co-culture and increased progressively thereafter. Endo-β-1,4-glucanase (EG) exhibited the highest activity, reaching a maximum of 377,516.82 ± 158,742 U/mL on day 19 (Figure 5(B2)). As an endoglucanase, EG cleaves internal β-1,4-glycosidic bonds within cellulose chains, thereby generating new chain ends for subsequent enzymatic hydrolysis.

Exo-β-1,4-cellobiohydrolase (CBH) and β-glucosidase (BG) activities were detected at comparable levels and also peaked on day 19 (Figure 5(B1,B3)). CBH sequentially releases cellobiose from the ends of cellulose chains, whereas BG hydrolyzes cellobiose into glucose. In contrast, polygalacturonase (PG), which primarily degrades pectin rather than cellulose, reached its highest activity on day 12 and declined after day 19 (Figure 5(B4)). This temporal pattern may indicate that pectic substrates were preferentially utilized during the earlier stages of colonization.

Ultimately, the transcriptional and biochemical results show that *S. commune* possesses the capacity to induce a coordinated cellulolytic enzyme system, comprising endoglucanase, cellobiohydrolase, and β-glucosidase, during its association with *D. nobile*. We therefore propose that fungal cell-wall-degrading enzymes may contribute to localized modification of host cell-wall components, which could participate in the activation of host JA-associated responses and metabolic reprogramming, ultimately promoting dendrobine accumulation. However, the direct causal role of individual cellulases in cell-wall remodeling and JA induction requires further experimental validation.

## 4. Discussion

### 4.1. S. commune Promotes Dendrobine Accumulation Through the Induction of Host Defense-Associated Metabolism

Endophytic fungi and their host plants engage in complex molecular and physiological interactions that have been shaped by long-term coevolution [40]. In some cases, endophytes possess biosynthetic gene clusters (BGCs) analogous to those of their hosts and can produce plant-associated specialized metabolites [41]. More commonly, however, endophytic fungi do not directly synthesize the corresponding host metabolites. Instead, they modulate host defense and metabolic networks, leading to enhanced biosynthesis and accumulation of plant-derived defense-related compounds [42]. Such microbe-mediated metabolic regulation has considerable potential for improving crop performance and enhancing the quality of medicinal plants.

Our previous analysis of the endophytic fungal community associated with wild-simulated *D. nobile*, combined with expression profiling of dendrobine-related genes, suggested that dendrobine accumulation is jointly influenced by host phytohormone signaling and endophytic fungal community composition [4]. In the present study, none of the 29 endophytic fungal isolates produced detectable dendrobine under the fermentation conditions tested (Supplementary Figure S1). This finding indicates that the increased dendrobine content observed in the symbiotic system is unlikely to result from direct fungal dendrobine production. Rather, it supports a model in which *S. commune* CY01-1H promotes dendrobine accumulation by altering host metabolic regulation.

Endophytic fungi may act as biological elicitors that activate or upregulate plant specialized-metabolic pathways. Fungal elicitors can include secreted metabolites, cell-wall fragments, and enzyme-mediated products released from plant tissues [43]. Following recognition by receptors at the plant cell surface, these signals can initiate complex signaling cascades that ultimately regulate the expression of pathway-specific genes and the activities of key metabolic enzymes. The resulting metabolic reprogramming may increase the accumulation of defense-associated specialized metabolites in the host. Our data indicate that this indirect, host-mediated mechanism is likely to underlie the promotion of dendrobine accumulation by *S. commune*.

### 4.2. S. commune-Mediated Enhancement of Dendrobine Accumulation Is Associated with JA Biosynthesis and a Symbiosis-Associated JA-Signaling State

Plants rely on phytohormone-mediated defense signaling to respond to microbial colonization and the associated competition for host-derived nutrients. JA is broadly distributed throughout the plant kingdom and regulates development, growth, and defense responses to biotic and abiotic challenges. It is therefore one of the principal phytohormones involved in plant responses to microbial association.

JA biosynthesis proceeds through the α-linolenic acid pathway (Supplementary Figure S4). Briefly, α-linolenic acid is released from chloroplast membrane lipids by phospholipases and is oxygenated by lipoxygenases (LOXs) to generate fatty-acid hydroperoxides, such as 13-hydroperoxyoctadecatrienoic acid (13-HPOT). These intermediates are subsequently converted by allene oxide synthase (AOS) and allene oxide cyclase (AOC) to 12-oxo-phytodienoic acid (OPDA). OPDA is transported into peroxisomes, where it is reduced by OPDA reductase 3 (OPR3) and subsequently subjected to three cycles of β-oxidation involving enzymes such as acyl-CoA oxidase (ACX), multifunctional protein (MFP), and 3-ketoacyl-CoA thiolase (KAT), ultimately yielding JA. JA can then be converted into bioactive derivatives, including jasmonoyl-isoleucine (JA-Ile) and MeJA [44]. LOXs are non-heme iron-containing dioxygenases that catalyze the oxygenation of polyunsaturated fatty acids containing a cis,cis-1,4-pentadiene moiety, generating conjugated fatty-acid hydroperoxides. These products serve as precursors for a range of bioactive oxylipins. LOXs are widely regarded as key early enzymes in JA biosynthesis [45,46]. In the present study, colonization by *S. commune* markedly increased the expression of Dn*LOX* genes, particularly Dn*LOX2* (Figure 3A), and significantly increased JA content in *D. nobile*. These results support activation of the α-linolenic acid–JA biosynthetic route during fungal colonization. Similar roles have been described in other plant species. In rice, Os*LOX9* is induced by *Magnaporthe oryzae* infection and contributes to JA biosynthesis; loss of Os*LOX9* reduces JA accumulation and increases disease susceptibility [44,47]. In maize, overexpression of *LOX4* promotes oxylipin production, upregulates JA-related genes, and enhances resistance to pathogens [48]. Notably, enhanced JA biosynthesis in SC-colonized plantlets was accompanied by an atypical downstream signaling profile. Although JA accumulation increased, expression of the JA–Ile conjugation-related genes *DnJAR1* and *DnJAR3* decreased, whereas genes encoding JAZ repressors were upregulated. JAZ proteins are central negative regulators of JA signaling. The observed transcriptional pattern therefore suggests that *S. commune* colonization activates JA biosynthesis while restraining sustained or excessive JA-responsive defense signaling. Such modulation may enable the host to maintain an appropriate level of defense preparedness without generating an immune response detrimental to fungal persistence. This interpretation is consistent with observations in other mutualistic plant–fungus systems. For example, the ectomycorrhizal fungus *Laccaria bicolor* can manipulate host JA signaling through interactions involving JAZ proteins, thereby facilitating compatible colonization [49]. Thus, *S. commune* may similarly reconfigure JA signaling in *D. nobile* to establish a balanced symbiotic state. In this state, JA-associated metabolic responses remain sufficiently active to promote dendrobine accumulation, whereas excessive immune activation is constrained. Nevertheless, direct functional evidence for the involvement of specific JAZ proteins in this interaction will be required to test this hypothesis.

### 4.3. Induction of Fungal Cellulolytic Enzymes May Contribute to Host Cell-Wall Remodeling and JA Activation

The plant cell wall is a dynamic and complex matrix composed principally of cellulose, hemicelluloses, pectins, lignin, suberin, and cell-wall-associated proteins. It represents a critical interface in host–microbe interactions [50]. To colonize plant tissues, microbes commonly deploy carbohydrate-active enzymes (CAZymes) that modify or degrade cell-wall polymers [51]. These enzymes include glycosyltransferases, polysaccharide lyases, glycoside hydrolases, and carbohydrate esterases [52]. In this study, dual RNA-seq revealed marked induction of *S. commune* genes involved in cellulose degradation during association with *D. nobile*. Consistent with the transcriptomic results, activities of endo-β-1,4-glucanase, cellobiohydrolase, and β-glucosidase increased during co-fermentation with *D. nobile* stem sections (Figure 5B). Together, these results demonstrate that *S. commune* activates a coordinated cellulolytic system in the presence of host tissues. This system may facilitate localized modification of host cell-wall polysaccharides during fungal establishment.

Cell-wall hydrolysis can release oligosaccharides that function as damage-associated molecular patterns (DAMPs). These molecules can activate plant immune signaling, including JA- and ethylene-dependent responses. This possibility is consistent with a previous study showing that α-mannosidase ShAM1, secreted by the rice endophyte *Streptomyces hygroscopicus* OsiSh-2, hydrolyzes rice cell-wall components and releases oligosaccharide signals that activate JA/ethylene-dependent immunity and enhance resistance to rice blast disease [53]. In the present study, endo-β-1,4-glucanase displayed the highest activity during the interaction. Because endoglucanases cleave internal β-1,4-glycosidic bonds, their activity could generate soluble cellooligosaccharides and new cellulose-chain ends. These products may function not only as carbon sources for the fungus but also as potential elicitors of host defense-associated signaling [54].

## 5. Conclusions

In this study, endophytic fungi were isolated from *D. nobile* stems, and *S. commune* CY01-1H was identified as the most effective isolate for enhancing dendrobine accumulation in *D. nobile* plantlets. Unlike many previously reported *D. nobile*-associated endophytes, which are predominantly ascomycetous fungi, SC is a basidiomycete, expanding the known diversity of fungal symbionts with potential applications in *D. nobile* cultivation.

The established *S. commune*-*D. nobile* symbiotic system enabled the simultaneous investigation of transcriptional responses in both partners by dual RNA-seq. In the host, SC colonization activated the α-linolenic acid–JA biosynthetic pathway, prominently increasing Dn*LOX2* expression and JA accumulation. However, downstream JA signaling showed a reprogrammed profile characterized by reduced Dn*JAR* expression and increased Dn*JAZ* expression, suggesting the establishment of a balanced symbiosis-associated defense state rather than a sustained, high-intensity immune response. This JA-associated reprogramming was accompanied by increased dendrobine accumulation.

In the fungal partner, host association induced the expression of genes encoding endoglucanases, cellobiohydrolases, and β-glucosidases, and the corresponding extracellular enzyme activities increased dynamically during interaction with *D. nobile* stem tissues. These findings support a working model in which *S. commune*-associated activation of a coordinated cellulolytic enzyme system may contribute to localized host cell-wall remodeling, thereby participating in the activation of host JA-associated metabolic responses and dendrobine accumulation.

Overall, this work identifies *S. commune* CY01-1H as a promising microbial biostimulant for improving dendrobine accumulation in *D. nobile*. It also provides a framework for understanding how fungal cell-wall-modifying activities and host hormone signaling may be coordinated during endophyte-mediated regulation of specialized metabolism. Further functional studies are needed to define the specific fungal enzymes, cell-wall-derived signals, and host signaling components responsible for this cross-kingdom interaction.

## Figures and Tables

**Table 1:** Grouping of fungal isolates for fermentation assays.

Groups	Strain	Phylum
1	*Arcopilus aureus*, *Aspergillus flavipes*, *Canariomyces arenarius*, *Clonostachys* sp., *Colletotrichum cordylinicola*, *Colletotrichum gloeosporioides*	Ascomycota
2	*Colletotrichum tropicicola*, *Fusarium concentricum*, *Fusarium proliferatum*, *Fusarium* sp., *Muyocopron alcornii*, *Penicillium oxalicum*	Ascomycota
3	*Preussia* sp., *Colletotrichum gloeosporioides*, *Diaporthe phaseolorum*, *Trichoderma guizhouense*, *Alternaria alternata*, *Nigrospora sphaerica*	Ascomycota
4	*Fusarium oxysporum*, *Hypoxylon fragiforme*, *Botryosphaeria dothidea*, *Xylariaceae* sp., *Shiraia bambusicola*, *Curvularia lunata*, *Penicillium* sp.	Ascomycota
5	*Bjerkandera adusta*, *Schizophyllum commune*, *Trametes hirsute*, *Perenniporia* sp.	Basidiomycota

**Table 2:** Taxonomic identification of 29 endophytic fungi isolated and purified from the stems of *D. nobile*.

No	Species	Genus	Family	Order	Class	Phylum
1	*Arcopilus aureus*	Arcopilus	Chaetomiaceae	Sordariales	Sordariomycetes	Ascomycota
2	*Aspergillus flavipes*	Aspergillus	Aspergillaceae	Eurotiales	Eurotiomycetes	Ascomycota
3	*Bjerkandera adusta*	Bjerkandera	Phanerochaetaceae	Polyporales	Agaricomycetes	Basidiomycota
4	*Canariomyces arenarius*	Canariomyces	Chaetomiaceae	Sordariales	Sordariomycetes	Ascomycota
5	*Clonostachys* sp.	Clonostachys	Bionectriaceae	Hypocreales	Sordariomycetes	Ascomycota
6	*Colletotrichum cordylinicola*	Colletotrichum	Glomerellaceae	Glomerellales	Sordariomycetes	Ascomycota
7	*Colletotrichum gloeosporioides*	Colletotrichum	Glomerellaceae	Glomerellales	Sordariomycetes	Ascomycota
8	*Colletotrichum tropicicola*	Colletotrichum	Glomerellaceae	Glomerellales	Sordariomycetes	Ascomycota
9	*Fusarium concentricum*	Fusarium	Nectriaceae	Hypocreales	Sordariomycetes	Ascomycota
10	*Fusarium proliferatum*	Fusarium	Nectriaceae	Hypocreales	Sordariomycetes	Ascomycota
11	*Fusarium* sp.	Fusarium	Nectriaceae	Hypocreales	Sordariomycetes	Ascomycota
12	*Muyocopron alcornii*	Muyocopron	Muyocopronales	Muyocopronales	Dothideomycetes	Ascomycota
13	*Penicillium oxalicum*	Penicillium	Aspergillaceae	Eurotiales	Eurotiomycetes	Ascomycota
14	*Preussia* sp.	Preussia	Sporormiaceae	Pleosporales	Dothideomycetes	Ascomycota
15	*Schizophyllum commune*	Schizophyllum	Schizophyllaceae	Agaricales	Agaricomycetes	Basidiomycota
16	*Trametes hirsute*	Trametes	Polyporaceae	Polyporales	Agaricomycetes	Basidiomycota
17	*Colletotrichum gloeosporioides*	Colletotrichum	Glomerellaceae	Glomerellales	Sordariomycetes	Ascomycota
18	*Diaporthe phaseolorum*	Diaporthe	Diaporthaceae	Diaporthales	Sordariomycetes	Ascomycota
19	*Trichoderma guizhouense*	Trichoderma	Hypocreaceae	Hypocreales	Sordariomycetes	Ascomycota
20	*Alternaria alternata*	Alternaria	Pleosporaceae	Pleosporales	Dothideomycetes	Ascomycota
21	*Nigrospora sphaerica*	Nigrospora	Pleosporaceae	Pleosporales	Dothideomycetes	Ascomycota
22	*Fusarium oxysporum*	Fusarium	Nectriaceae	Hypocreales	Sordariomycetes	Ascomycota
23	*Hypoxylon fragiforme*	Hypoxylon	Hypoxylaceae	Xylariales	Sordariomycetes	Ascomycota
24	*Botryosphaeria dothidea*	Botryosphaeria	Botryosphaeriaceae	Botryosphaeriales	Dothideomycetes	Ascomycota
25	*Xylariaceae* sp.	Xylariaceae	Xylariaceae	Xylariales	Sordariomycetes	Ascomycota
26	*Perenniporia* sp.	Perenniporia	Polyporaceae	Polyporales	Agaricomycetes	Basidiomycota
27	*Shiraia bambusicola*	Shiraia	Shiraiaceae	Pleosporales	Dothideomycetes	Ascomycota
28	*Curvularia lunata*	Curvularia	Pleosporaceae	Pleosporales	Dothideomycetes	Ascomycota
29	*Penicillium* sp.	Penicillium	Aspergillaceae	Eurotiales	Eurotiomycetes	Ascomycota

**Table 3:** The literature search summarized the interactions between the 29 endophytic fungal strains and their host plants.

Specific Name	Host Plant	Interactions	
*Arcopilus aureus*	*Cucumis melo* L. Grapevines	Causes muskmelon leaf spot disease. Pigment produced	[22,23]
*Aspergillus flavipes*	Bananas (*Musa* spp.)	Biocontrol of leaf blight disease	[24]
*Bjerkandera adusta*	*Brassica napus* L. Wheat	Inhibits the growth of *Fusarium oxysporum* Inhibits the growth of *Fusarium graminearum* and biocontrol against wheat scab	[15,16]
*Canariomyces arenarius*	Black and red goji berries	NO	[25]
*Clonostachys* sp.	Rice and *Arabidopsis thaliana*	enhanced the growth of both rice and *A. thaliana.*	[26]
*Muyocopron alcornii*	*Hevea brasiliensis*	A symbiotic relationship of potential endophytic	[27]
*Penicillium oxalicum*	barley	mediated phosphate solubilization, enhancing barley growth	[28]
*Preussia* sp.	Rice *Glycyrrhiza uralensis*	producing nitric oxide, gibberellins, and indole acetic acid and improving rice plant growth. Positively affected the total biomass, root biomass, and active ingredient content of host plants under drought stress.	[17,18]
*Schizophyllum commune*	*Panax ginseng* *Perilla frutescens* Blueberry	Enhances hairy roots growth and ginsenoside biosynthesis. Enhancing their antioxidant capacity A potential biocontrol agent against blueberry root rot.	[12,13,29]
*Trametes hirsute*	*Avocado trees* Rice	Pathogen of white rot. Directly promotes growth by releasing VOCs.	[30,31]
*Diaporthe phaseolorum*	*Combretum lanceolatum*	Endophyte fungi	[32]
*Trichoderma guizhouense*	*Camellia oleifera* Maize Watermelon	Biocontrol agent against anthracnose. Promotes seed germination, enhances plant growth, and improves rhizosphere soil quality.	[19,20]
*Alternaria alternata*	*Chrysanthemum* *morifolium*	Enhances the immune defense response to improve resistance.	[33]
*Hypoxylon fragiforme*	*Acai palm*	Inhibits plant anthracnose	[21]
*Botryosphaeria dothidea*	*Rosa roxburghii*	Causes leaf spot disease	[34]
*Xylariaceae* sp.	*Mangifera indica* L.	Causing stem end rot	[35]
*Perenniporia* sp.	Hardwood Trees	By secreting carbohydrate-active enzymes (CAzymes) to degrade components of plant cell walls	[36]
*Shiraia bambusicola*	Bamboos	No	[37]
*Penicillium* sp.	Citrus	Penicillanic acid can serve as an alternative option for the prevention and control of citrus canker.	[38]

## Data Availability

Data is contained within the article or Supplementary Material.

## Supplementary Materials

The following supporting information can be downloaded at: https://www.mdpi.com/article/10.3390/biom16101533/s1, Figure S1: Gas chromatogram of 29 strains of endophytic fungi grouped and mixed after fermentation mycelium and fermentation liquid; Figure S2: Total ion chromatograms and mass spectra of dendrobine standard and representative sample; Figure S3: MRM chromatograms of jasmonic acid standard and a representative sample; Figure S4: Biosynthetic pathway of jasmonic acid; Table S1: Sequences of primers used in this study.

## Abbreviations

The following abbreviations are used in this manuscript:

*D. nobile*	*Dendrobium nobile*
MeJA	methyl jasmonate
SHAM	salicylhydroxamic acid
*SC*	*S. commune*
PDA	potato dextrose agar plates
PDB	potato dextrose broth
JA	Jasmonic acid
