# Supplementary material for: Schizophyllum commune Enhances Dendrobine Accumulation in Dendrobium nobile in Association with Induced Fungal Cellulolytic Activity and Host Jasmonate Biosynthesis
Source: Biomolecules. 2026 Oct 20;16(10):1533. doi: 10.3390/biom16101533 (PMC13702697; doi:10.3390/biom16101533)
Supplement: Supplementary file 1 [file biomolecules-16-01533-s001.zip › Figure S1.pdf]

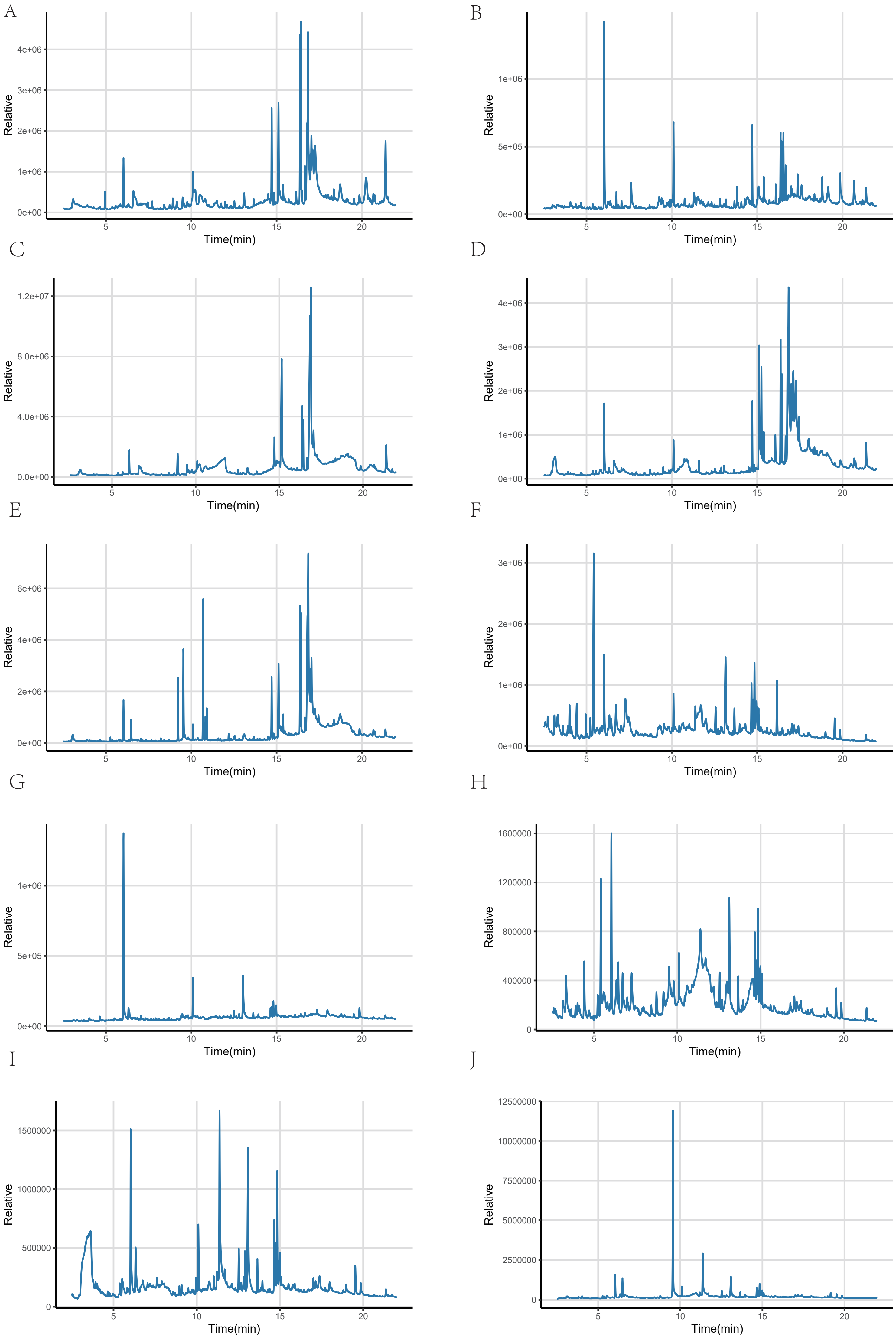

Gas chromatogram of 29 strains of endophytic fungi grouped and mixed after fermentation mycelium and fermentation liquid (Figures A-E show the mycelium and Figures F-J show the fermented liquid)
