# Supplementary material for: Schizophyllum commune Enhances Dendrobine Accumulation in Dendrobium nobile in Association with Induced Fungal Cellulolytic Activity and Host Jasmonate Biosynthesis
Source: Biomolecules. 2026 Oct 20;16(10):1533. doi: 10.3390/biom16101533 (PMC13702697; doi:10.3390/biom16101533)

MRM chromatogram of the Jasmonic acid standard

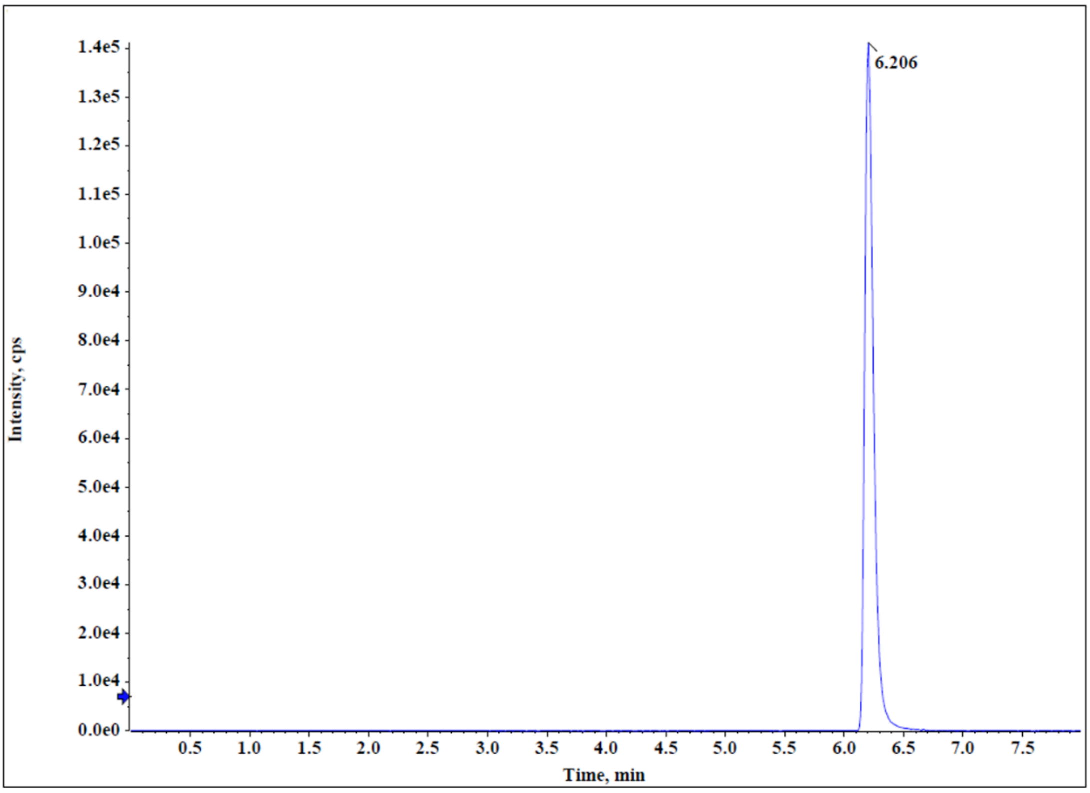

MRM chromatogram of one sample

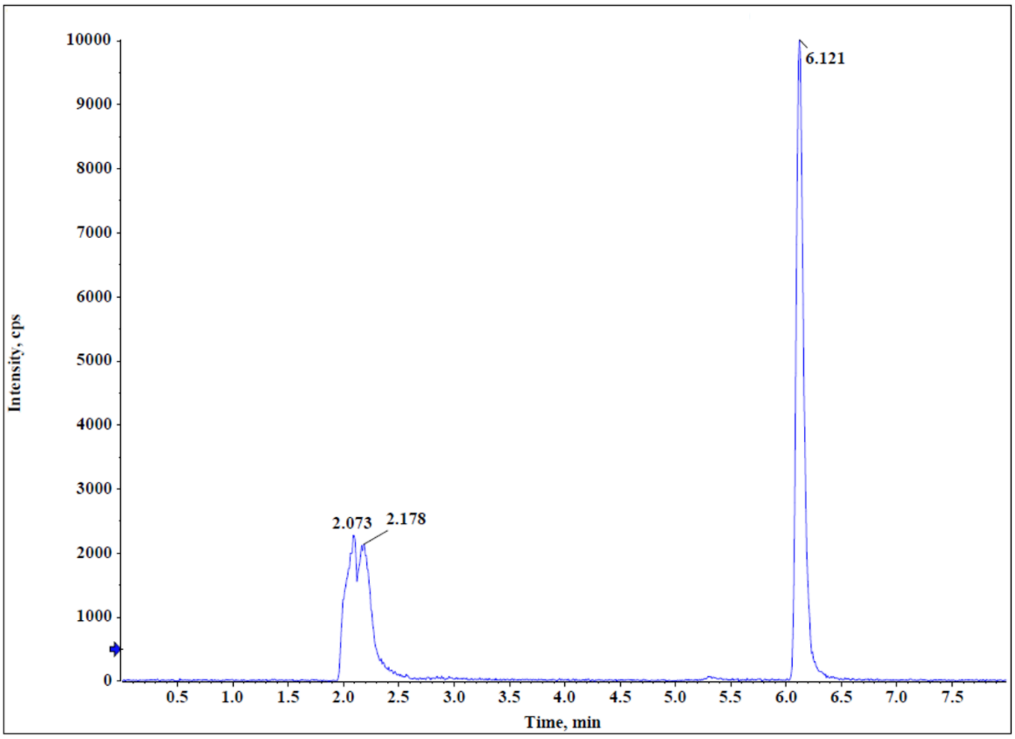

Supplement: Supplementary file 1 [file biomolecules-16-01533-s001.zip › Figure S3.pdf]
