# Supplementary material for: Schizophyllum commune Enhances Dendrobine Accumulation in Dendrobium nobile in Association with Induced Fungal Cellulolytic Activity and Host Jasmonate Biosynthesis
Source: Biomolecules. 2026 Oct 20;16(10):1533. doi: 10.3390/biom16101533 (PMC13702697; doi:10.3390/biom16101533)
Supplement: Supplementary file 1 [file biomolecules-16-01533-s001.zip › Table S1.pdf]

Supplementary Table S1. Sequences of primers used in this study

Genes	Forward Primer(5' to 3')	Reverse Primer(5' to 3')
KFK09_024652（DnLox1）	TCGCTAACATCCTCACCA	TCTCAATCTCCTCCTCCG
KFK09_024653（DnLox2）	TCCACCTTCATCTTCCCC	CTTCCTTGCTCGTTTTCG
KFK09_026255（DnJAR1）	TCCTCCTGTTAATCCCTC	TACATTTGTCGTCGCCGT
KFK09_002180（DnJAR3）	TTCGCTTAGTTCGGGGAC	GAAAAATGGCTTGTCTGT
KFK09_005063（DnJAR4）	GCTACCACAGCCACTACA	AGCATATCAAACCACATA
KFK09_021993（DnJAZ1）	AAAGCATCACTCCATCGC	TCCTTCACAGAAACAGCA
KFK09_024707(DnJAZ2)	CCGTCCGTCCAAACTCTG	GATGAACCTAAACCCCCA
